# Povidone-Iodine-Induced Acute Kidney Injury in a 23-Year-Old Woman: The First Clinical Case Report From the Republic of Cyprus

**DOI:** 10.7759/cureus.24034

**Published:** 2022-04-11

**Authors:** Panagiotis Papadopoulos, Stelios Iordanou, Fotini Georgiou, Dimitris Kalifatidis, Elena Herodotou, Chrystalla Timiliotou-Matsentidou

**Affiliations:** 1 Intensive Care Unit, Limassol General Hospital, State Health Services Organisation (SHSO), Limassol, CYP

**Keywords:** hydrotubation, anuria, povidone-iodine, acute kidney injury, aki

## Abstract

Povidone-iodine (PI) is considered a generally safe broad-spectrum, antiseptic substance, which is widely used in healthcare services, mostly for burn patients, in wound treatment, surgical wound irrigation, as well as for a few gynecological indications. Although it is safe, its use on mucosa can cause toxicity due to iodine absorption and the high concentration in the serum. PI toxicity is absorption depended and has been associated with severe adverse events including acute kidney injury (AKI). To our knowledge, there are only three cases of PI-induced AKI after uterine instillation in the published literature. We report a case of severe PI-induced AKI that occurred in a 23-year-old female due to iodine systemic absorption immediately post uterine instillation, in terms of infertility evaluation.

The patient was admitted to the intensive care unit (ICU), supported with mechanical ventilation and treated with renal replacement therapy (RRT). Renal function and urine output improved and the patient was discharged from ICU.

Severe adverse events may be linked to internal use, therefore, PI on mucosa should be used with extreme caution. Clinicians should be aware of iodine intoxication and possible AKI.

## Introduction

Povidone-iodine (PI) is a broad-spectrum antiseptic substance that exhibits a strong antimicrobial action and is widely used in healthcare services [1,2]. It is safe for use in combination of substances, and it is on the list of Essential Medicines of the World Health Organization [3]. Despite that it is considered to be safe, PI has been associated with severe adverse events, such as iodine-induced hypothyroidism or hyperthyroidism, and acute kidney injury (AKI) [4-7] due to secondary iodine mucus absorption and the presence of high levels in serum. Kidneys play a significant role in the clearance of iodine, therefore levels above their clearance capabilities can cause intoxication.

We report a case of a young adult woman who developed PI-induced AKI, after a diagnostic hysteroscopy and laparoscopy procedure in terms of infertility evaluation. To our knowledge, this is the first case of PI-induced AKI in the Republic of Cyprus, and one of the few [8-10] in the published literature associated with a gynecology procedure.

## Case presentation

A 23-year-old woman was admitted electively to a private gynecology clinic for evaluation of infertility. She was found to have bilateral hydrosalpinx and partial uterine septum and she was scheduled for diagnostic hysteroscopy and laparoscopy. Her previous medical history was unremarkable, apart from a BMI of 28 and a reported allergy to penicillin. The operation was uneventful, lasting approximately 150 mins; transcervical resection of the uterine septum and reopening of the hydrosalpinx bilaterally were performed, as well as lysis of a few adnexal adhesions. During the procedure, 50 ml of diluted PI 1% (10% solution, diluted 1:10) were instilled through the uterine to check fallopian tube patency, a technique called dye hydrotubation, with no signs of adverse or allergic reactions perioperatively. The patient returned to the postoperative ward. Antibiotic prophylaxis was provided as per the clinic’s protocol, as well as postoperative analgesia and intravenous fluids.

Eight hours postoperatively the patient became oliguric. Despite aggressive fluid challenges, she became anuric overnight. Laboratory testing on the next morning revealed: WCC 46 x 10^3^/μL, haemoglobin 12.2 g/dl, platelet count 206 x 10^3^/μL, creatinine 2.5 mg/dl (no renal function tests performed pre-operatively), Potassium level 5.1 mmol/L. Her clotting was grossly normal [International Normalized Ratio (INR) 1.32, Activated Partial Thromboplastin Time (aPTT) 37.4 sec] and fibrinogen levels were measured at 292 mg/dl (Table 1).

**Table 1:** Summary table of patient’s full blood count, biochemistry and clotting test results during the first nine days of her ICU hospitalization. WBC: White blood cells, NEUT: Neutrophils, LYM: Lymphocytes, MONO: Monocytes, EOS: Eosinophils, BASO: Basophils, IG: Immature Granulocytes, RBC: Red Blood Cells, HGB: Hemoglobin, HCT: Hematocrit, MCV: Mean Corpuscular Volume, MCH: Mean Cell Hemoglobin, PLT: Platelets, MPV: Mean Platelet Volume, LDH: Lactate dehydrogenase, CRP: C-reactive protein, PT: Prothrombin Time, INR: International Normalized Ratio, APTT: Activated Partial Thromboplastin Time, TBIL: Total bilirubin, ALP: Alkaline phosphatase, γ-GT: Gamma-glutamyl Transferase, ALT, SGPT: Alanine aminotransferase, AST, SGOT: Aspartate aminotransferase, L: Litre

Laboratory	ICU Admission Day	DAY 1	DAY 2	DAY 3	DAY 4	DAY 5	DAY 6	DAY 7	DAY 8	DAY 9
WBC (10^3^/μL)	45.06	36.00	21.33	16.58	16.02	15.56	14.64	11.55	13.71	18.91
NEUT (%)	95.8	87.9	78.1	67.5	69.4	67.4	61.1	63.4	77.4	77.3
LYM (%)	2.3	8.5	13.6	7.5	10.9	13.9	17.6	20.8	11.9	13.6
MONO (%)	1.8	3.4	4.6	7.5	10.2	10.5	10.9	10.9	8.9	8.0
EOS (%)	0.0	0.1	3.5	8.2	9.1	7.8	9.9	4.6	1.4	0.8
BASO (%)	0.1	0.1	0.2	0.4	0.4	0.4	0.5	0.3	0.4	0.3
IG (%)	0.9	0.8	0.9	1	1.4	1.7	2.4	1.7	1.2	0.8
NEUT (10^3^/μL)	43.19	31.66	16.65	11.19	11.11	10.48	8.95	7.33	10.62	14.60
LYM (10^3^/μL)	1.03	3.05	2.90	2.72	1.75	2.16	2.57	2.40	1.63	2.58
MONO (10^3^/μL)	0.79	1.22	0.99	1.24	1.64	1.63	1.60	1.26	1.22	1.51
EOS (10^3^/μL)	0.01	0.04	0.74	1.36	1.46	1.22	1.45	0.53	0.19	0.16
BASO (10^3^/μL)	0.04	0.03	0.05	0.07	0.06	0.07	0.07	0.03	0.05	0.06
IG (10^3^/μL)	0.42	0.27	0.19	0.16	0.23	0.26	0.35	0.20	0.16	0.16
RBC (10^3^/μL)	3.67	3.13	2.63	2.69	2.79	2.72	2.68	2.58	2.60	2.60
HGB (g/dL)	10.4	8.9	7.5	7.7	8.1	7.8	7.7	7.4	7.4	7.4
HCT (%)	31.6	26.8	22.7	23.7	24.5	24	23.9	22.8	23.2	23.4
MCV (fL)	86.1	85.6	86.3	88.1	87.8	88.7	89.2	88.4	89.2	90
MCH (pg)	28.3	28.4	28.5	28.6	29	28.7	28.7	28.7	28.5	28.5
PLT (10^3^/μL)	190	133	88	109	117	160	232	304	360	537
MPV (fL)	11.1	10.9	10.2	10.1	9.6	10.2	9.9	9.4	9.1	9.6
CRP (mg/L)	123	107.96	85.61	84.05	91.62	89.26	63.58		28.32	36.97
LDH	3319	2104	1172	792	634	590	575		729	740
TBIL (mg/dL)	0.97	0.60			0.72				0.50	0.55
ALP (U/L)	159	78	57		63	63	13		71	76
γ-GT (U/L)	22	16	14		40	33		34	49	47
ALT, SGPT (U/L)	25	36	49		26	19		14	40	21
AST, SGOT (U/L)	124	69	40		18	15	13		45	27
Urea (mg/dL)	79	51	22	18	25	32	26		42	83
Creatinine	3.04	2.02	1.26	1.25	1.34	1.64	1.41		2.03	4.07
Fibrinogen (mg/dL)	292	302	306		473		447			467
PT (sec)	14.9	13	13.3	12.9	12.6	13.9	12.1			13.3
INR	1.36	1.14	1.16	1.13	1.10	1.23	1.05			1.17
APTT (sec)	37.62	38.71	40.51	41.39	37.19	40.34	39.69			42.83

The arterial blood gases showed fully compensated metabolic acidosis (pH 7.42, HCO3 16.1 mmol/L, pCO2 29 mmHg) and lactate levels <1.0 mmol/L. On the same morning, after informed consent was obtained, the patient underwent emergency diagnostic laparoscopy, under the joint care of the gynecologist and the general surgeon. The gastrointestinal tract was found intact, but the uterine showed signs of diffuse acute inflammation with no ischaemic changes or perforation.

**Figure 1:**
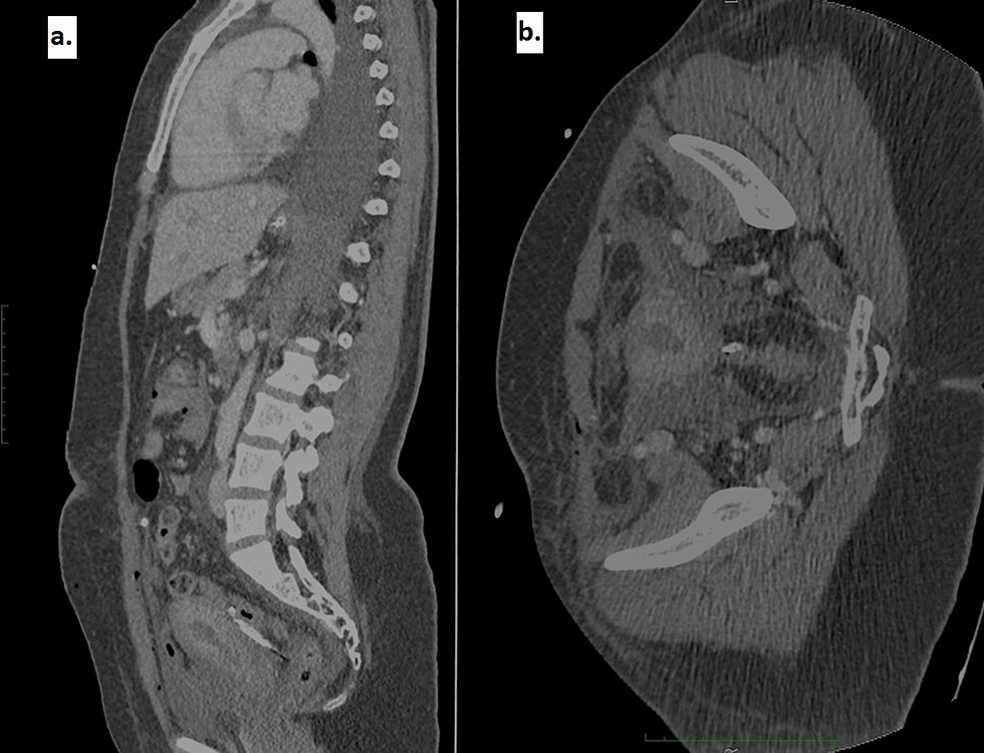
Computed tomography (CT) scan of the chest and abdomen-pelvis CT showed heterogeneous uterine density and moderate diffuse endometrial thickening.

The patient was transferred to our hospital immediately postoperatively, while still intubated, sedated and mechanically ventilated, and was admitted to our intensive care unit. Her arterial blood gas showed a strong metabolic acidosis with a pH of 7.21, pCO2 43.9 mmHg, bicarbonate level of 15.0 mmol/L, base excess -10.0 mmol/L, potassium 5.2 mmol/L, lactate 1.8 mmol/L. She remained under full sedation, her PaO2/FiO2 was more than 300 on FiO2 0.30, with no need for vasopressor support, hemodynamically stable, and afebrile. Her SAPS II score was 45. Her blood count was: WCC 45 x 10^3^/μL (polymorphonuclear cells 95%), haemoglobin 10.4 g/dl, platelet count 190 x 10^3^/μL (Table 1). The peripheral blood smear did not show fragmented red cells. Her LDH was 3319 units/L, bilirubin 0.97 mg/dl, alkaline phosphatase 159 units/L, and renal parameters were further deranged: urea 79 mg/dl, creatinine 3.04 mg/dl, phosphate 8.0 mg/dl, total calcium 7.5 mg/dl with albumin levels of 2.6 mg/dl. C3 and C4 complement levels were within normal limits. Her CRP was raised to 125 mg/L and her procalcitonin level was negative (semiquantitative method). A small sample of urine was obtained and microscopic evaluation revealed numerous red blood cells with only a few hemoglobin casts, 2-3 white cells per high power field, and few epithelial cells. Meropenem, vancomycin, and Vibramycin were given to cover for the possibility of sepsis and pelvic inflammatory disease, and swabs from the cervix and vagina were obtained for culture. A CT scan of the chest and abdomen-pelvis showed heterogeneous uterine density, moderate diffuse endometrial thickening, and a small accumulation of free fluid in the pelvis (Figure 1).

The possibility of iodine systemic absorption and intoxication was raised and continuous venovenous hemodialysis (CVVHD) with citrate for anticoagulation was initiated, 6 hours post ICU admission and a total of 32 hours after instillation of povidone-iodine. The following settings were used: blood flow 160 ml/min, dialysate flow rate 40 ml/kg/hr. She was also treated with prednisone 1 mg/kg/day to attenuate any potential acute interstitial nephritis development.

On the second day of ICU admission, a serum sample was sent to an external laboratory abroad (France) in order to measure iodine levels since there is no availability in the Republic of Cyprus. Thus, the sample was taken approximately 16 hours after initiation of renal replacement treatment. The result - retrospectively - showed levels of 136,900 mcg/L (normal range 40-100 mcg/L), which confirmed the diagnosis of povidone-iodine intoxication. Eosinophil count raised from zero to 1,360 x 10^3^/μL on the third day after admission. Transvaginal ultrasound showed normal uterine thickness and no signs of ischemia or perforation.

**Figure 2:**
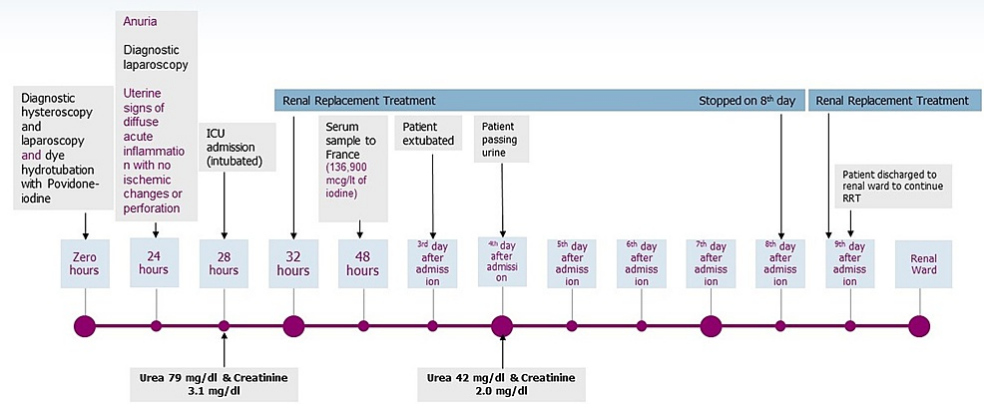
Patient clinical course

The patient was extubated on day 3, as she has been quite restless with difficulty weaning on previous attempts. She started urinating on day 4, while CVVHD was stopped on the 8th day of the ICU stay. She was discharged to the renal ward on the 9th day of hospitalization, oliguric (Day 8: urea 42 mg/dl, creatinine 2.03 mg/dl), with a plan to continue on regular hemodialysis as required. She regained full kidney function 20 days later after ICU discharge. The patient's clinical course is shown in Figure 2. Cultures that had been previously obtained revealed no pathogen and antimicrobial treatment was stepped down to meropenem monotherapy.

## Discussion

PI exhibits strong bactericidal and fungicidal activity through various mechanisms. It causes cell wall pore formation of microorganisms and disturbs solid-liquid interfaces in the lipid membranes, eventually leading to cytolysis. It reacts with unsaturated fatty acids of cell membranes [1,2], but also reacts with starch and glycogen to produce characteristic blue-black or brown-black complexes and oxidizes amino acids and nucleotides [11]. In addition, it has been suggested that it is more potent as an antiviral agent compared to other commonly used antiseptics [12].

Its use is common practice as a skin disinfectant, especially in surgery preparation, but it is also being used for burn patients, in deep wound treatment, for surgical wound irrigation [13-19], for intracavity lavage in abdominal operations [20,21] as well as for chemical pleurodesis [22] and mediastinal irrigation in cases of mediastinitis [4,5,23,24]. It has also been used for recurrent lymphocele sclerosis [6] and a couple of gynecological indications [7-10], like in our case, as a dye for imaging hydrotubation. More recently, it has been tested as a potential anti-cancer agent against various cell lines in vivo, including malignant pleural mesothelioma, colorectal cancer, breast and lung carcinoma, and melanoma [25-28]. Accordingly, in the clinical setting, it has been applied with intrapleural administration for thymic epithelial tumors and malignant pleural mesothelioma [29-31]. Despite its beneficial effects, iodine’s systemic absorption has the potential to cause significant adverse effects or even death if multiorgan dysfunction occurs.

Absorption has been observed in every route including intact skin, but it dramatically increases when used internally, therefore rapid systemic absorption is not surprising to this or any other patient. Toxicity and AKI have been reported post mediastinal [4,5,23,24], wound, subcutaneous or burn irrigation [13-18], lymphocele irrigation after renal transplant [6], and also after skin application in infants or oral ingestion [32-34]. Experimental data raise concern that iodine toxicity could follow peritoneal lavage in peritonitis, although this has not been reported in humans [35,36]. Moreover, toxicity pertains to acute kidney injury, presenting with metabolic acidosis, hyperosmolality, and hypernatraemia [6,8-10,16,23,33]. Despite that PI-induced AKI is a common cause of in-hospital renal dysfunction, its pathophysiology has not yet been understood completely [37].

The concentration of the solution used plays a significant role as well, to avoid iodine intoxication. Absorption into the blood follows Fick's law of diffusion, *“the rate of diffusion of a substance across a unit area (such as a surface or membrane) is proportional to the concentration gradient” *[38]. Nevertheless, the appropriate use has proven to be clinically safe when used in a concentration of 1% internally (peritoneum irrigation) [20,39].

To our knowledge, there are three cases similar to ours, in the literature, with systemic absorption emerging immediately post uterine instillation [8-10], reported in 2006, 2007, and 2011.

The first case [8] refers to a 41-year-old woman who underwent hydrotubation with the use of PI as a dye and exhibited AKI due to intoxication. She was treated with renal replacement therapy (RRT) and iodemia was reduced over nine days of treatment. The second case [9] refers to a 36-year-old woman who underwent uterine opacification before laparotomy and salpingotomy. She developed AKI and was treated with RRT for more than 20 days. The last case refers to a 37-year-old woman [10] who underwent hysteroscopy with the use of PI as the contrast agent. She developed AKI and was treated with diuretics and RRT. All patients' medical history was unremarkable. All three cases are similar to our case report in terms of surgical procedure, and patient management.

Clinicians should be aware that, the route, the concentration used as well as the underlying renal function are important factors in PI intoxication and possible AKI.

## Conclusions

We report the clinical course and management of a 23-year-old woman who developed PI-induced AKI, after diagnostic hysteroscopy and laparoscopy in terms of evaluation of infertility. The current report illustrates that severe adverse events may be linked to the internal use of PI, therefore, its use on mucosa should be used with extreme caution since an overabsorption of iodine can result in toxic serum levels and subsequently AKI.

Moreover, prompt RRT initiation in the presence of PI-induced AKΙ may result in serum iodine clearance, prevent furthermore kidney damage, and contribute to reverse kidney function.
